# Patient-Reported Testing Burden of Breast Magnetic Resonance Imaging Among Women With Ductal Carcinoma In Situ: An Ancillary Study of the ECOG-ACRIN Cancer Research Group (E4112)

**DOI:** 10.1001/jamanetworkopen.2021.29697

**Published:** 2021-11-02

**Authors:** Soudabeh Fazeli, Bradley S. Snyder, Ilana F. Gareen, Constance D. Lehman, Seema A. Khan, Justin Romanoff, Constantine A. Gatsonis, Kathy D. Miller, Joseph A. Sparano, Christopher E. Comstock, Lynne I. Wagner, Ruth C. Carlos

**Affiliations:** 1 Department of Radiology, University of California, San Diego; 2 Center for Statistical Sciences, Brown University School of Public Health, Providence, Rhode Island; 3 Department of Epidemiology, Brown University School of Public Health, Providence, Rhode Island; 4 Department of Radiology, Massachusetts General Hospital, Harvard Medical School, Boston; 5 Department of Medicine, Northwestern University Feinberg School of Medicine, Chicago, Illinois; 6 Department of Surgery, Indiana University, Indianapolis; 7 Albert Einstein College of Medicine, Montefiore Medical Center, Bronx, New York; 8 Department of Radiology, Memorial Sloan-Kettering Cancer Center, New York, New York; 9 Wake Forest School of Medicine, Wake Forest Baptist Comprehensive Cancer Center, Winston-Salem, North Carolina; 10 Department of Radiology, University of Michigan, Ann Arbor; 11 Program for Women’s Health Effectiveness Research, University of Michigan, Ann Arbor; 12 Institute for Health Policy and Innovation, University of Michigan, Ann Arbor

## Abstract

**Importance:**

The use of magnetic resonance imaging (MRI) in pretreatment planning of ductal carcinoma in situ (DCIS) remains controversial. Understanding changes in short-term health-related quality of life associated with breast MRI would allow for a more complete comparative effectiveness assessment.

**Objective:**

To assess whether there are changes in patient-reported quality of life associated with breast MRI among women diagnosed with DCIS.

**Design, Setting, and Participants:**

This cohort study was a substudy of a nonrandomized clinical trial conducted at 75 participating US institutions from March 2015 to April 2016. Women recently diagnosed with unilateral DCIS who were eligible for wide local excision and had a diagnostic mammogram within 3 months of study registration were included. A total of 355 women met the eligibility criteria and underwent the study MRI. Data analysis was performed from June 3, 2020, to July 1, 2021.

**Exposures:**

Participants underwent bilateral breast MRI within 30 days of study registration and before surgery. Information on patient-reported testing burden for breast MRI was collected after MRI and before surgery.

**Main Outcomes and Measures:**

The primary outcome of this substudy was the patient-reported testing burden of breast MRI, measured by the Testing Morbidities Index (TMI) summated scale score. The TMI is a 7-item instrument that evaluates the temporary changes in quality of life associated with imaging before, during, and after the test (0 represents the worst possible, 100 the hypothetical ideal test experience).

**Results:**

Of the 355 women who met the eligibility criteria, 244 (69%) completed both questionnaires and were included in this analysis. The median age was 59 years (range, 34-85 years). The mean MRI TMI summated scale score was 85.9 (95% CI, 84.6-87.3). Of the 244 women, 142 (58%) experienced at least some fear and anxiety before the examination, and 120 women (49%) experienced fear and anxiety during the examination. A total of 156 women (64%) experienced pain or discomfort during the examination. In multivariable analyses, greater test-related burden was associated with higher levels of cancer worry (regression coefficient, −2.75; SE, 0.94; *P* = .004).

**Conclusions and Relevance:**

In this cohort study, a clinically meaningful breast MRI testing burden among women with DCIS was revealed that was significantly associated with cancer worry. Understanding the potential quality-of-life reduction associated with MRI, especially when used in combination with mammography, may allow development of targeted interventions to improve the patient experience.

## Key Points

**Question:**

Is there a short-term reduction in health-related quality of life associated with breast magnetic resonance imaging (MRI) in patients with ductal carcinoma in situ?

**Findings:**

In this cohort study of 244 women diagnosed with ductal carcinoma in situ, a short-term reduction in quality of life associated with MRI was revealed, primarily owing to fear before the test and fear and physical discomfort during the test.

**Meaning:**

Understanding the potential reduction in quality of life associated with MRI in patients with ductal carcinoma in situ may allow development of targeted interventions to improve the patient’s experience.

## Teaser

This cohort study examines short-term patient-reported changes in health-related quality of life in women with ductal carcinoma in situ undergoing magnetic resonance imaging for treatment planning.

## Introduction

The term “scanxiety” describes the temporary distress and decrease in health-related quality of life (HRQOL) associated with diagnostic testing.^1^ Although diagnostic tests, such as breast magnetic resonance imaging (MRI), are presumed to primarily affect patients’ health by guiding clinical decision-making and management, testing can have additional emotional and physical effects.^2^ Health-related quality of life has been defined as the extent to which a disease and its treatment affects a patient’s sense of overall function and well-being.^3^ Thus, the emotional and physical effects of imaging are salient components of HRQOL among patients with cancer undergoing diagnostic testing. Presumed morbidity associated with these tests is particularly important for patients with cancer, who are at increased risk for negative emotional outcomes, including fear of cancer recurrence.^4^

Breast MRI has emerged as a more sensitive modality for detecting ductal carcinoma in situ (DCIS) compared with mammography,^5^ offering the potential to better inform surgical planning. However, testing-related HRQOL reduction represents a risk associated with breast MRI used for DCIS detection and characterization. The Testing Morbidities Index (TMI), a 7-item instrument, evaluates the temporary HRQOL changes associated with imaging before, during, and after the test.^6^ This measure allows for formal assessment of patient preferences and comparison between different tests.^7^ The TMI has been used for patients undergoing breast biopsy,^7^ colonoscopy,^7^ and pelvic MRI^8^; however, limited data exist on the testing burden associated with breast MRI among patients with DCIS.

Furthermore, prior assessments of diagnostic testing–related fear and anxiety compared individual tests.^7,8^ However, in clinical practice, we use multiple tests in sequence (ie, the diagnostic pathway). For patients with DCIS, diagnostic mammography and breast MRI are often combined. Therefore, evaluation of the HRQOL reduction associated with MRI must quantify the cumulative burden when added to mammography.

The purposes of this cohort study were to (1) assess the patient-reported changes in HRQOL—specifically the potential testing burden—associated with breast MRI using the TMI among women diagnosed with unilateral DCIS who were eligible for wide local excision; (2) assess the association between prespecified covariates, including sociodemographic characteristics and cancer worry, and breast MRI testing burden; and (3) quantify the cumulative testing burden of a DCIS diagnostic pathway including diagnostic mammography and breast MRI.

## Methods

This cohort study was approved by the National Cancer Institute, Division of Cancer Prevention and by the local institutional review board at each participating site. Written informed consent was obtained from all participants.^9^ This study followed the Strengthening the Reporting of Observational Studies in Epidemiology (STROBE) reporting guideline.^10^

### Data and Sample

The present study was an ancillary study to a prospective nonrandomized clinical trial coordinated by the Eastern Cooperative Oncology Group–American College of Radiology Imaging Network (ECOG-ACRIN) Cancer Research Group (E4112) that enrolled women with unilateral DCIS without microinvasive or invasive disease determined by core biopsy who were candidates for wide local excision from 75 US institutions between March 2015 and April 2016. Primary results and details on trial design and eligibility criteria are described elsewhere.^11^ Study collection of demographic variables, including self-identified race and ethnicity, were required by the National Cancer Institute, Division of Cancer Prevention; however, participants were not required to respond to any of the demographic questions. Other race included American Indian/Alaska Native, Asian, multiple races reported, not reported, and Unknown. For brevity, we refer to self-reported race and ethnicity as race and ethnicity.

Participants were required to undergo diagnostic mammography of the affected breast within 3 months before study registration. Once enrolled, participants underwent bilateral breast MRI before surgery. Patient-reported outcome measures assessed cancer worry, decision autonomy preference, HRQOL, and testing burden for mammography and breast MRI. Information on patient-reported testing burden for mammography was scheduled to be collected within 2 weeks after study registration and before the breast MRI (time point T0). Information on patient-reported testing burden for breast MRI was scheduled to be collected after MRI and before surgery (time point T1).

### Patient-Reported Outcome Data Collection

**Figure 1.**
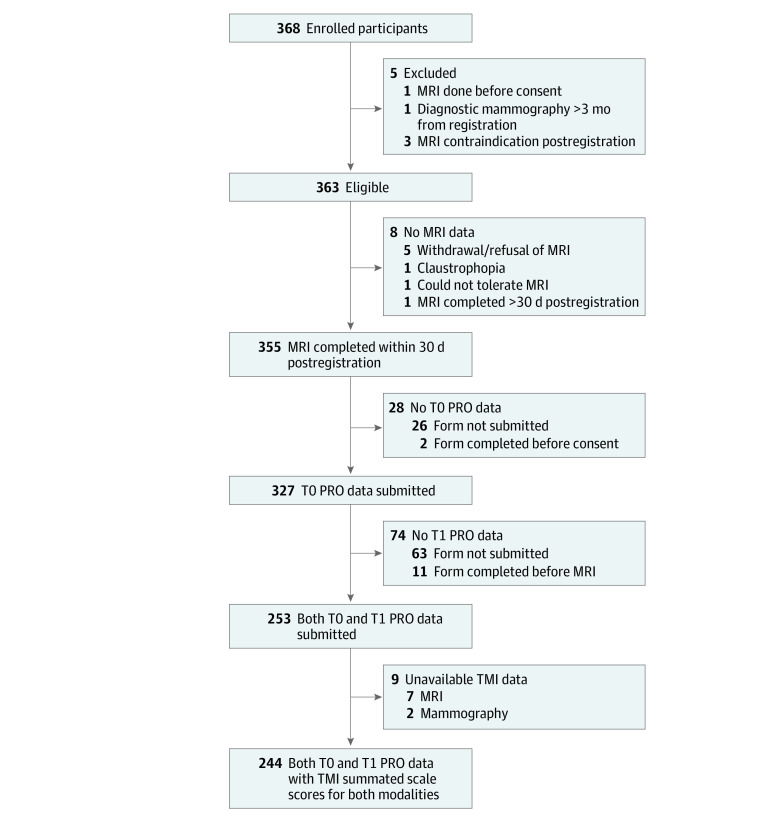
Study Flow Diagram and Schema Questionnaire Administration MRI indicates magnetic resonance imaging; PRO, patient-reported outcome; and TMI, Testing Morbidities Index.

At study registration, women opted to complete questionnaires online via an email prompt or by postal mail. Patients who did not respond received follow-up emails and/or phone calls. Participants who completed the study MRI and both T0 and T1 patient-reported outcome questionnaires were included in this substudy (Figure 1).

### Measures and Outcomes

The primary outcome of this substudy was the patient-reported testing burden of diagnostic mammography and breast MRI as measured by TMI summated scale scores. Components of TMI are grouped as experienced during preparation for the test (pain/discomfort or fear), during the test (intraprocedural pain/discomfort, embarrassment, or fear), and immediately after the test (temporary mental or physical discomfort) (eMethods in the Supplement). We used a modified TMI, with item response collected using a 4-point Likert scale rather than a 5-point Likert scale. The total score was then converted to a 0 to 100 scale,^6,12^ with 0 representing the worst possible and 100 the hypothetical ideal test experience. Secondary outcomes included the TMI domain-level score for each of the 7 survey items and the TMI component scores calculated separately before, during, and after the testing experience. Details on the calculation of the TMI summated scale score and component scores are given in the eMethods in the Supplement.

Prespecified independent variables included cancer worry, decision autonomy preference, HRQOL, age, race, ethnicity, and insurance status. Cancer worry information was collected at T0 and measured using the 3-item cancer worry subscale of the Assessment of Survivor Concerns.^13^ Each Assessment of Survivor Concerns item has a 4-category response scale of 1 (not at all), 2 (a little bit), 3 (somewhat), and 4 (very much). The mean of the 3 cancer worry items (fear of cancer recurrence, new cancer diagnosis, and diagnostic tests) was determined for each participant, arriving at a semicontinuous measure ranging from 1 to 4, in which higher values indicate higher levels of cancer worry. Decision autonomy preference information was collected at T0 and was measured using the Control Preferences Scale,^14^ which assesses patient decision involvement in treatment choice. The Control Preferences Scale consists of a single item on a 5-point scale, typically reduced to a 3-category scale (patient-based, shared, and surgeon-based) for analysis. Information on HRQOL was collected at T0 and was measured using the Patient-Reported Outcomes Measurement Information System-10, a 10-item questionnaire addressing global physical and mental health,^15^ with the raw scores converted to mental and physical T scores. T-score distributions are standardized such that a score of 50 represents the mean for the US general population, and the SD around that mean is 10 points. Higher scores represent better HRQOL.

### Statistical Analysis

Statistical analysis was conducted from June 3, 2020, to July 1, 2021. Descriptive statistics for demographic and clinical characteristics were calculated to describe the analysis cohort. Excluded participants were compared with analyzable participants using the *t* test or nonparametric Wilcoxon rank sum test for continuous variables and the exact version of the χ^2^ test for categorical variables. Breast MRI TMI summated scale scores are reported for the analysis cohort. In addition, the various component scores (before, during, and after the examination) were compared using the paired *t* test. A post hoc multiplicity adjustment with the Holms-Bonferroni method^16^ was used to control the familywise error rate for the 3 between-component comparisons.

Analyses were also conducted to examine potential associations between the breast MRI TMI summated scale score and prespecified participant characteristics. Univariable associations were examined using linear regression for continuous covariates and 1-way analysis of variance for categorical covariates. A multivariable linear regression model was then fit. The coefficient of determination was reported for each regression model.

To address the cumulative burden of breast MRI after diagnostic mammography, we used the additive method of estimating joint utility.^17^ Details on the calculation of the joint utility score are given in the eMethods in the Supplement. Univariable associations with the same prespecified covariates were examined using linear regression for continuous covariates and 1-way analysis of variance for categorical covariates. Sensitivity analyses were conducted using the multiplicative and minimum methods of estimating joint utility.^17^ Multivariable linear regression models were then fit for each joint utility score.

Additional sensitivity analyses were conducted using multiple imputation by chained equations^18^ to assess the potential influence of missing covariate data on the multivariable models (eMethods in the Supplement). Data were analyzed using SAS, version 9.4 (SAS Institute Inc) and R, version 4.0.4 (R Foundation for Statistical Computing). All reported *P* values are 2-sided, with the significance threshold set at .05.

## Results

### Sample Characteristics

Figure 1 shows the study flow diagram. Of the 368 participants enrolled, 355 met eligibility criteria and underwent the study MRI. The substudy included 244 women (69%) who completed both T0 and T1 questionnaires; the median age was 59 years (range, 34-85 years). Table 1 summarizes other demographic and clinical characteristics of included and excluded participants. Among women included in the study, fewer were Black/African American (30 of 53 [57%]) or other race (17 of 31 [55%]) compared with White (197 of 271 [73%]), Hispanic (9 of 21 [43%]) compared with non-Hispanic or unknown (235 of 334 [70%]), and insured by Medicaid or uninsured (8 of 18 [44%]) compared with insured (188 of 273 [69%]). The median time between the preregistration diagnostic mammogram and the corresponding TMI assessment was 28 days (IQR, 18-39.5 days), and for the breast MRI and TMI assessment, 20.5 days (IQR, 11-38 days).

**Table 1.** Sociodemographic and Clinical Characteristics of the Study Cohort

Characteristic	Eligible participants with study MRI, No. (N = 355)	Participants^a^		*P* value^b^
		Included (n = 244)	Excluded (n = 111)	
Age, median (range), y	59 (34-87)	59 (34-85)	59 (35-87)	.39
Race				
Black/African American	53	30 (57)	23 (43)	.02
White	271	197 (73)	74 (27)	
Other^c^	31	17 (55)	14 (45)	
Ethnicity				
Hispanic	21	9 (43)	12 (57)	.01
Non-Hispanic or unknown	334	235 (70)	99 (30)	
Insurance status				
Private insurance	273	188 (69)	85 (31)	.04
Medicare or other government insurance	64	48 (75)	16 (25)	
Medicaid or uninsured	18	8 (44)	10 (56)	
DCIS grade				
Low	58	39 (67)	19 (33)	.58
Intermediate	144	95 (66)	49 (34)	
High	140	102 (73)	38 (27)	
Unknown	13	8 (62)	5 (38)	
DCIS longest diameter, median (IQR), mm^d^	11 (6-20)	11.5 (6-22)	11 (8-15)	.54^e^
Decision autonomy preference				
My surgeon should make the decision with little input from me	3	2 (67)	1 (33)	.39^e^
My surgeon should make the decision but seriously consider my opinion	23	19 (83)	4 (17)	
My surgeon and I should make the decision together	230	174 (76)	56 (24)	
I should make the decision after seriously considering my surgeon’s opinion	73	49 (67)	24 (33)	
Missing	26	0	26 (100)	
Diagnostic mammography TMI summated scale score, median (range)^f^	90.5 (42.9-100.0)	90.5 (42.9-100.0)	90.5 (52.4-100.0)	.59
ASC Cancer Worry subscale score, median (range)^g^	2.3 (1.0-4.0)	2.0 (1.0-4.0)	2.3 (1.0-4.0)	.19^e^
PROMIS-10 score, median (range)^h^				
Physical T score	50.8 (26.7-67.7)	54.1 (26.7-67.7)	50.8 (29.6-67.7)	.08^e^
Mental T score	50.8 (31.3-67.6)	50.8 (31.3-67.6)	50.8 (38.8-67.6)	.18^e^

Abbreviations: ASC, Assessment of Survivor Concerns; DCIS, ductal carcinoma in situ; MRI, magnetic resonance imaging; PROMIS-10, Patient-Reported Outcomes Measurement Information System–10; TMI, Testing Morbidities Index.

^a^ Data are presented as number (percentage) of participants unless otherwise indicated. Percentages correspond to rows.

^b^ *P* values compare participants in the analysis set vs those excluded. For continuous variables, the *P* value corresponds to the *t* test or the nonparametric Wilcoxon rank sum test as appropriate. For categorical variables, the *P* value corresponds to the exact version of the χ^2^ test.

^c^ American Indian/Alaska Native, Asian, multiple races reported, not reported, and Unknown.

^d^ As reported on the diagnostic mammogram.

^e^ The *P* value for the comparison was performed after removing missing values.

^f^ The TMI is a 7-item instrument that evaluates the temporary changes in quality of life before, during, and after a test (0 represents the worst possible and 100 the hypothetical ideal test experience).

^g^ Each ASC item has a 4-category response scale of 1 (not at all), 2 (a little bit), 3 (somewhat), and 4 (very much). The mean of the 3 cancer worry items (fear of cancer recurrence, new cancer diagnosis, and diagnostic tests) was determined for each participant, arriving at a semicontinuous measure ranging from 1 to 4, in which higher values indicate higher levels of cancer worry.

^h^ A 10-item questionnaire addressing global physical and mental health. Raw scores are converted to mental and physical T scores. T score distributions are standardized such that a score of 50 represents the mean for the US general population, and the SD around that mean is 10 points. Higher scores represent better HRQOL.

### Patient-Reported Diagnostic Testing Burden of Breast MRI

Table 2 summarizes the breast MRI TMI domain-level, component, and summated scale scores. Of 244 women, 142 (58%) experienced at least some fear and anxiety before the examination, and 120 women (49%) experienced fear and anxiety during the examination. In contrast, 79 women (32%) reported at least some pain or discomfort before the examination, and 156 women (64%) experienced pain or discomfort during the examination. After the examination, 210 women (86%) reported no residual mental discomfort and 212 (87%) reported no physical discomfort. The mean TMI summated scale score was 85.9 (95% CI, 84.6-87.3). The before-examination component (82.0; 95% CI, 79.9-84.0; *P* < .001) and during-examination component (82.7; 95% CI, 80.9-84.4; *P* < .001) scores were both significantly lower than the after-examination component score (94.8; 95% CI, 93.3-96.3; difference between before and after examinations: 12.8; 95% CI, 10.5-15.2; difference between during and after examinations: 12.2; 95% CI, 10.1-14.2).

**Table 2.** Proportion of Patients Experiencing Testing Burden Based on Testing Morbidities Index Domain-Level Scores

Domain level	Patients, No. (%)
**Before MRI**	
Pain/discomfort	
None	165 (68)
Some	73 (30)
A lot	5 (2)
Extreme	1 (0.4)
Fear or anxiety	
None	102 (42)
Some	113 (46)
A lot	22 (9)
Extreme	7 (3)
**During MRI**	
Pain/discomfort	
None	88 (36)
Some	132 (54)
A lot	19 (8)
Extreme	5 (2)
Embarrassment	
None	185 (76)
Some	59 (24)
A lot	0
Extreme	0
Fear or anxiety	
None	124 (51)
Some	106 (43)
A lot	11 (5)
Extreme	3 (1)
**After MRI**	
Mental discomfort	
None	210 (86)
Some	30 (12)
A lot	4 (2)
Extreme	0
Physical discomfort	
None	212 (87)
Some	28 (11)
A lot	2 (1)
Extreme	2 (1)

Abbreviation: MRI, magnetic resonance imaging.

In univariable analyses, higher (better) breast MRI TMI summated scale scores were significantly associated with decreased cancer worry (regression coefficient, −2.79; SE, 0.82; *P* < .001), higher (better) physical T scores (regression coefficient, 0.20; SE, 0.09; *P* = .03) and mental T scores (regression coefficient, 0.32; SE, 0.10; *P* = .002), and older age (regression coefficient, 0.13; SE, 0.07; *P* = .049) (eTable 1 in the Supplement). In multivariable analyses, the association between cancer worry and the breast MRI TMI summated scale score persisted after adjustment for potential confounders (regression coefficient, −2.75; SE, 0.94; *P* = .004) (Table 3). This association persisted in sensitivity analyses using multiple imputation to adjust for missing covariate data (Table 3). After controlling for demographic characteristics and patient-reported outcomes, Black/African American women reported significantly worse MRI testing burden compared with White women (regression coefficient, −4.18; SE, 2.10; *P* = .048); however, this difference did not hold in sensitivity analyses incorporating adjustment for missing data using multiple imputation (Table 3). No other variables remained significantly associated after covariate adjustment. Although significant associations were identified, the combined covariates explained only 15% of the variation in breast MRI TMI summated scale scores (*R*^2^ = 0.15) (Table 3).

**Table 3.** Multivariable Regression Models for the Breast MRI TMI Summated Scale Score and the Joint Utility Score of a Testing Sequence of Diagnostic Mammography Followed by Breast MRI

Variable	Breast MRI TMI summated scale score model^a^				Joint utility score model^a^			
	Complete case estimates (n = 206)^b^		Multiple imputation estimates (n = 244)^c^^,^^d^		Complete case estimates (n = 206)^e^		Multiple imputation estimates (n = 244)^c^^,^^f^	
	Parameter estimate (SE)	*P* value	Parameter estimate (SE)	*P* value	Parameter estimate (SE)	*P* value	Parameter estimate (SE)	*P* value
Intercept^g^	83.58 (1.69)	<.001	84.11 (1.59)	<.001	74.19 (2.36)	<.001	74.18 (2.29)	<.001
Age	0.10 (0.08)	.24	0.05 (0.08)	.56	0.05 (0.12)	.67	0.06 (0.11)	.61
Revised 5-item ASC Cancer Worry subscale	−2.75 (0.94)	.004	−2.28 (0.90)	.01	−6.77 (1.31)	<.001	−6.27 (1.31)	<.001
PROMIS-10								
Physical T score	0.10 (0.11)	.37	0.10 (0.11)	.34	0.27 (0.15)	.07	0.32 (0.18)	.08
Mental T score	0.21 (0.13)	.11	0.19 (0.13)	.16	0.17 (0.18)	.34	0.11 (0.22)	.63
Race								
Black/African American (vs White)	−4.18 (2.10)	.048	−3.11 (2.06)	.13	−3.50 (2.94)	.23	−1.71 (2.96)	.56
Other (vs White)	−1.22 (2.88)	.67	−1.43 (2.84)	.61	−0.27 (4.02)	.95	−0.61 (4.08)	.88
Ethnicity								
Hispanic (vs Non-Hispanic or Unknown)	2.74 (3.93)	.49	0.64 (3.77)	.87	−0.64 (5.49)	.91	−0.59 (5.40)	.91
Insurance status								
Medicare or other government insurance vs private insurance	−0.41 (2.03)	.84	0.35 (1.92)	.85	1.19 (2.83)	.67	0.67 (2.76)	.81
Medicaid or uninsured vs private insurance	0.64 (3.76)	.86	1.15 (3.86)	.77	1.25 (5.25)	.81	1.06 (5.53)	.85
Decision autonomy preference								
Surgeon-selected (vs patient-selected)	1.92 (2.86)	.50	1.93 (2.78)	.49	−1.15 (3.99)	.77	−1.03 (3.99)	.80
Shared (vs patient-selected)	3.39 (1.83)	.06	2.87 (1.71)	.10	2.30 (2.55)	.37	2.84 (2.46)	.25

Abbreviations: ASC, Assessment of Survivor Concerns; MRI, magnetic resonance imaging; PROMIS-10, Patient-Reported Outcomes Measurement Information System–10; TMI, Testing Morbidities Index.

^a^ Parameter estimates for continuous covariates are interpreted as the change in mean response per 1-unit increase. Parameter estimates for categorical covariates are interpreted as the difference in mean response in comparison with the reference level.

^b^ *R*^2^ = 0.15 from the multivariable linear regression model.

^c^ Multiple imputation for missing covariate data was performed for the subset of patients with available TMI summated scale scores for both modalities (n = 244) (Figure 1).

^d^ *R*^2^ = 0.11 from the multiply imputed multivariable linear regression model.

^e^ *R*^2^ = 0.20 from the multiply imputed multivariable linear regression model.

^f^ *R*^2^ = 0.17 from the multivariable linear regression model.

^g^ Continuous covariates were centered. Thus, the intercept can be interpreted as the mean MRI TMI summated scale score (or joint utility score) for patients who are at the mean age (59.1 years), mean ASC cancer worry level (2.41), mean physical (52.40) and mental (51.76) T scores, and who are at the reference level of the categorical covariates (White, non-Hispanic, private insurance, and patient-selected decision preference).

### Joint Utility Estimates

The mean TMI summated scale score for diagnostic mammography before breast MRI was 90.0 (95% CI, 88.9-91.0). With the use of the additive method for estimating joint utility, a pathway of diagnostic mammography followed by breast MRI after DCIS diagnosis yielded a mean joint utility score of 75.9 (95% CI, 73.9-77.9), corresponding to a 15.7% increase in testing burden over mammography alone.

**Figure 2.**
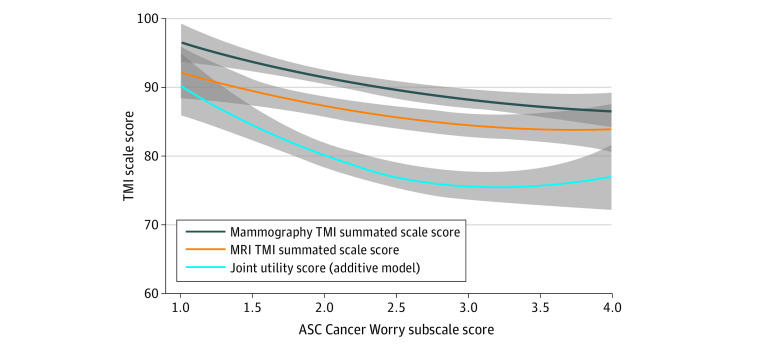
Testing Burden Associated With Cancer Worry A LOESS smoother was used to plot the diagnostic mammography Testing Morbidities Index (TMI) summated scale score, the magnetic resonance imaging (MRI) TMI summated scale score, and the joint utility score of a diagnostic pathway combining both tests as estimated by the additive model. ASC indicates Assessment of Survivor Concerns.

In univariable analyses, higher (better) joint utility scores were associated with decreased cancer worry (regression coefficient, −6.6; SE, 1.2; *P* < .001) (Figure 2), higher (better) physical T scores (regression coefficient, 0.37; SE, 0.13; *P* = .006) and mental T scores (regression coefficient, 0.52; SE, 0.15; *P* < .001), and older age (regression coefficient, 0.22; SE, 0.10; *P* = .03) (eTable 2 in the Supplement). In multivariable analyses, only cancer worry was associated with the joint utility score (regression coefficient, −6.77; SE, 1.31; *P* < .001) (Table 3). This association persisted in sensitivity analyses using multiple imputation to adjust for missing covariate data (regression coefficient, −6.27; SE, 1.31; *P* < .001) (Table 3). Regardless of the model used for estimating the cumulative testing burden, cancer worry was significantly associated with the joint utility score (eTable 3 and eTable 4 in the Supplement).

## Discussion

This study showed a clinically meaningful patient-reported breast MRI testing burden, commonly defined as an effect size equal to one-third to one-half of the SD.^19^ Patient anxiety before the test and anxiety and physical discomfort during the test were associated with the TMI summated scale scores. A diagnostic pathway with breast MRI after diagnostic mammography for DCIS increased the testing burden by 15.7% compared with mammography alone. Cancer worry was significantly associated with greater breast MRI testing burden and greater cumulative burden of a mammography and breast MRI diagnostic pathway.

Quality-of-life measurements are typically applied to long-term health states or conditions.^6^ However, individuals place value even on temporary events, including burden associated with diagnostic testing.^6^ The use of MRI in pretreatment planning for patients with DCIS remains controversial. In a previous publication from the E4112 trial including patients with DCIS who were eligible for breast conservation surgery and underwent MRI after diagnostic mammography, treatment for 19.2% was converted to mastectomy.^11^ Among these patients, treatment for 38.5% was converted to mastectomy owing to the MRI findings. Understanding the HRQOL reduction associated with breast MRI may allow for a more complete assessment of the comparative effectiveness of a breast MRI diagnostic pathway for patients with DCIS.

Testing is not a benign procedure and may result in fear and anxiety (ie, “scanxiety”) before and during the testing process that are unrelated to the underlying diagnosis or treatment. Other studies evaluated imaging-associated distress using the Impact of Events Scale, which was developed to assess symptoms indicating posttraumatic stress disorder.^20,21^ In a small cross-sectional study^20^ of recurrent or metastatic non–small cell lung cancer, patients who underwent recent imaging reported moderate anxiety. A prospective study of coronary computed tomographic angiography showed mild test-related anxiety.^21^ Our results advance previous findings by measuring testing burden using a scale specifically developed to assess the burden of diagnostic imaging tests. Furthermore, we measured the full experience associated with testing, including temporal effects and isolation of anxiety from physical discomfort.

We analyzed the components of testing burden from the individual domains that make up the TMI, such as pain or discomfort before the test, fear or embarrassment during the test, and physical or mental function after the test. Knowledge of domain-level outcomes can help with identification and implementation of appropriate targeted interventions to improve the test-related experience. Women undergoing breast MRI after diagnostic mammography for DCIS may benefit from preprocedural education and counseling to reduce anticipatory stress and improve overall testing experience.^22^

Although an association between Black/African American race (vs White race) and increased testing burden was detected among included participants, this association did not persist after sensitivity analyses accounting for missing data. Future studies are required to better assess any disparities in testing burden based on race and ethnicity.

Among the sample of women, higher testing burden was associated with greater levels of cancer worry, a proxy for fear of cancer recurrence, which is one of the most distressing consequences of cancer.^23^ Women with higher levels of fear of cancer recurrence experience more worry about their diagnostic test results, leading to poorer testing experiences. Targeted interventions to mitigate fear of cancer and recurrence^24^ early after the diagnosis may improve HRQOL outcomes including diagnostic testing burden.

In univariable analysis, younger age was associated with higher test-related burden. These findings may be in part attributable to the association between age, HRQOL, and fear of cancer recurrence.^25^ Younger cancer survivors may perceive their cancer as more unexpected and generally report higher levels of anxiety and depression.^25^ Associations between poorer HRQOL and fear of recurrence have been previously reported.^4^

To our knowledge, this study represents the first evaluation of the use of TMI for testing burden associated with breast MRI among women with newly diagnosed DCIS. Sakala et al^8^ reported TMI summated scale scores among women undergoing pelvic MRI for pelvic pain that were comparable to our results. In an active surveillance population with prostate cancer, reported TMI summated scale scores for men undergoing prostate MRI were greater than those in our study,^26^ potentially owing to differences in participants’ sex and age as well as MRI techniques.

### Strengths and Limitations

This study has strengths. The sample size was large compared with previous studies using TMI. We used a scale specifically developed to assess imaging-related burden, used a prospective study design, and enrolled patients from multiple sites, including community practices and academic centers.

The study also has limitations. The population was largely composed of White women and was limited to women recently diagnosed with DCIS who were awaiting treatment. Thus, our results may not be generalizable to all racial groups, women without cancer, or women with invasive breast cancer. As with all patient-reported outcome evaluations, responses were subject to recall bias that may have been influenced by the duration between the test and the TMI measurement or TMI measurement and receipt of the MRI result; the severity of testing burden may also influence recall bias independent of this duration. The mode of survey administration may represent another potential limitation, although participants were given the choice of mode and were presumed to select the one most likely to ensure survey completion. In addition, only 69% of patients who received the study MRI completed both the T0 and T1 patient-reported outcomes and were included in our analysis, potentially resulting in inadvertent selection bias owing to nonresponse.

## Conclusions

In this study, breast MRI for the evaluation of DCIS was associated with fear and anxiety among women with DCIS, particularly before and during the test. Preprocedural interventions to manage test expectations and mitigate cancer worry may improve the MRI testing experience. In the absence of guidelines supporting the use of MRI in the diagnostic pathway for DCIS, women may be exposed to additional, previously unquantified harm. Although this evaluation focused on women with DCIS, breast MRI use continues to increase, for example, in enhanced screening among women with dense breasts^27^ or diagnostic workup of abnormal screening mammography findings.^28^ This increased use presents an opportunity for future assessment of breast MRI test burden and targeted interventions to reduce MRI-related fear and anxiety in a broader population.

## Notes

Supplement. **eMethods.** Detailed Methods **eTable 1.** Summary of Univariable Associations Between the Breast MRI TMI Summated Scale Score and Prespecified Covariates **eTable 2.** Summary of Univariable Associations Between the Joint Utility Scores and Prespecified Covariates **eTable 3.** Multivariable Regression Model for the Joint Utility Score (Multiplicative Model) of a Testing Sequence of Diagnostic Mammography Followed by Breast MRI in DCIS **eTable 4.** Multivariable Regression Model for the Joint Utility Score (Minimum Model) of a Testing Sequence of Diagnostic Mammography Followed by Breast MRI in DCIS
eReferences
