# Errors in Table 3 Footnotes

**DOI:** 10.1001/jamanetworkopen.2021.41485

**Published:** 2021-12-06

In the Original Investigation titled “Patient-Reported Testing Burden of Breast Magnetic Resonance Imaging Among Women With Ductal Carcinoma In Situ: An Ancillary Study of the ECOG-ACRIN Cancer Research Group (E4112),”^1^ published November 2, 2021, in Table 3, footnote e was corrected to ^“^*R*^2^ = 0.20 from the multivariable linear regression model” and footnote f was corrected to “*R*^2^ = 0.17 from the multiply imputed multivariable linear regression model.”
