# Supplementary material for: Patient-Reported Testing Burden of Breast Magnetic Resonance Imaging Among Women With Ductal Carcinoma In Situ: An Ancillary Study of the ECOG-ACRIN Cancer Research Group (E4112)
Source: JAMA Netw Open. 2021 Nov 2;4(11):e2129697. doi: 10.1001/jamanetworkopen.2021.29697 (PMC8564581; doi:10.1001/jamanetworkopen.2021.29697)
Supplement: Supplement. — eMethods. Detailed Methods eTable 1. Summary of Univariable Associations Between the Breast MRI TMI Summated Scale Score and Prespecified Covariates eTable 2. Summary of Univariable Associations Between the Joint Utility Scores and Prespecified Covariates eTable 3. Multivariable Regression Model for the Joint Utility Score (Multiplicative Model) of a Testing Sequence of Diagnostic Mammography Followed by Breast MRI in DCIS eTable 4. Multivariable Regression Model for the Joint Utility Score (Minimum Model) of a Testing Sequence of Diagnostic Mammography Followed by Breast MRI in DCIS eReferences [file jamanetwopen-e2129697-s001.pdf]

Supplementary Online Content

Fazeli S, Snyder BS, Gareen IF, et al. Patient-reported testing burden of breast magnetic resonance imaging among women with ductal carcinoma in situ: an ancillary study of the ECOG-ACRIN Cancer Research Group (E4112). *JAMA Netw Open*. 2021;4(11):e2129697. doi:10.1001/jamanetworkopen.2021.29697

eMethods. Detailed Methods

eTable 1. Summary of Univariable Associations Between the Breast MRI TMI Summated Scale Score and Prespecified Covariates

eTable 2. Summary of Univariable Associations Between the Joint Utility Scores and Prespecified Covariates

eTable 3. Multivariable Regression Model for the Joint Utility Score (Multiplicative Model) of a Testing Sequence of Diagnostic Mammography Followed by Breast MRI in DCIS

eTable 4. Multivariable Regression Model for the Joint Utility Score (Minimum Model) of a Testing Sequence of Diagnostic Mammography Followed by Breast MRI in DCIS

eReferences

This supplementary material has been provided by the authors to give readers additional information about their work.

© 2021 Fazeli S et al. *JAMA Network Open.*

eMethods. Detailed Methods

1. Development of the TMI

Using the general techniques for development of a multi-attribute utility-based index, Swan et al developed the Testing Morbidities Index (TMI) by applying preference weights to a health classification for measuring short term health related quality of life related to diagnostic testing.[1] The conceptualization of the TMI is consistent with the wait-tradeoff (WTO) concept, since the burden of testing represents a toll on the process of medical care. WTO scaling for TMI allows the burden of the testing experience to be deductible from quality-adjusted life years and has been applied to a wide range of diagnostic tests [2–4]. The TMI surveys used for diagnostic mammogram and breast MRI are provided below.

Modified TMI questionnaire

The following questions are about how you felt before, during and after your mammogram used to diagnose DCIS. *Think only about that mammogram.*

Before the mammogram

1. Based on what you knew about the mammogram overall, how much fear did you have about having it beforehand?

- No fear at all
- Some fear
- A lot of fear
- Extreme fear

2. How much discomfort was involved with preparing for the mammogram overall (e.g., changing your diet, fasting, taking, or using drugs or other preparations that may have caused various side effects)?

- No pain or discomfort
- Some pain or discomfort
- A lot of pain or discomfort
- Extreme pain or discomfort

During the mammogram

3.Thinking about the mammogram, how much pain or discomfort did you experience while it was happening, overall?

- No pain or discomfort
- Some pain or discomfort
- A lot of pain or discomfort
- Extreme pain or discomfort

4. How much embarrassment overall, if any, did you feel during the mammogram experience (revealing parts of your body, movements of the body or other aspects that may have caused you to feel awkward or uncomfortable?)

- No embarrassment
- Some embarrassment
- A lot of embarrassment
- Extreme embarrassment

5. How much fear did you feel during this mammogram experience overall?

- No fear
- Some fear
- A lot of fear
- Extreme fear

After the mammogram

6. Thinking about your ability to do your daily activities after the mammogram, how serious overall were any temporary problems you had with your physical function (for example, working, walking, ability to move your back, neck and arms, or anything else because of pain, cramping, weakness, etc., that occurred)?

- No problems
- Some problems
- A lot of problems
- Extreme problems

7. Thinking about your ability to do your daily activities after the mammogram, how serious overall were any temporary problems you had with your mental function or state of mind (for example, fear, anxiety/worry, depression, difficulty concentrating, etc.)?

This may be difficult for you to separate from the difficulty of going through the whole process of being a patient, but, as best you can, try to focus on the particular problems that only the mammogram may have caused.

© 2021 Fazeli S et al. *JAMA Network Open.*

- No problems
- Some problems
- A lot of problems
- Extreme problems

The following questions are about how you felt before, during and after your MRI.
Before the MRI

1. Based on what you knew about the MRI overall, how much fear did you have about having it beforehand?
   - No fear at all
   - Some fear
   - A lot of fear
   - Extreme fear
2. How much discomfort was involved with preparing for the MRI overall (e.g., changing your diet, fasting, taking, or using drugs or other preparations that may have caused various side effects)?
   - No pain or discomfort
   - Some pain or discomfort
   - A lot of pain or discomfort
   - Extreme pain or discomfort

During the MRI

3. Thinking about the MRI, how much pain or discomfort did you experience while it was happening, overall?
   - No pain or discomfort
   - Some pain or discomfort
   - A lot of pain or discomfort
   - Extreme pain or discomfort
4. How much embarrassment overall, if any, did you feel during the MRI experience (revealing parts of your body, movements of the body or other aspects that may have caused you to feel awkward or uncomfortable?)
   - No embarrassment
   - Some embarrassment
   - A lot of embarrassment
   - Extreme embarrassment
5. How much fear did you feel during this MRI experience overall?
   - No fear
   - Some fear
   - A lot of fear
   - Extreme fear

After the MRI

6. Thinking about your ability to do your daily activities after the MRI, how serious overall were any temporary problems you had with your physical function (for example, working, walking, ability to move your back, neck and arms, or anything else because of pain, cramping, weakness, etc., that occurred)?
   - No problems
   - Some problems
   - A lot of problems
   - Extreme problems
7. Thinking about your ability to do your daily activities after the MRI, how serious overall were any temporary problems you had with your mental function or state of mind (for example, fear, anxiety/worry, depression, difficulty concentrating, etc.)?
   This may be difficult for you to separate from the difficulty of going through the whole process of being a patient, but, as best you can, try to focus on the particular problems that only the MRI may have caused.
   - No problems
   - Some problems
   - A lot of problems
   - Extreme problems

2. Calculation of the TMI summated scale scores

Computation of the TMI summated scale scores for both mammography and breast MRI follow Swan et al [1] (refer to Appendix B of that paper), with a modified 4-point Likert scale. Relevant data were collected at PRO time point T0 (baseline) for

© 2021 Fazeli S et al. *JAMA Network Open.*

mammography, and at PRO time point T1 (prior to surgery) for breast MRI. The seven TMI questions are shown in **Supplement 1**. For each question, the 4-point scale was coded as follows: None=1, Some=2, A lot=3, Extreme=4. Refer to each coded question as L_1, L_2, L_3, L_4, L_5, L_6, L_7. The TMI summated scale score (semi-continuous, range 0-100; 0=worst, 100=best) was then calculated for each modality as follows:

$$\text{TMI summated scale score} = 100 - \left[\frac{100}{7}\right] \sum_{i=1}^{7} \frac{Li-1}{3}$$

Component TMI scores were also computed for the before, during and after components, where the above equation was adjusted based on the number of questions in each component section, with the before component comprising the first two questions, the during component the middle three questions, and the after component the last two questions:

$$\text{TMI before component score} = 100 - \left[\frac{100}{2}\right] \sum_{i=1}^{2} \frac{Li-1}{3}$$

$$\text{TMI during component score} = 100 - \left[\frac{100}{3}\right] \sum_{i=1}^{3} \frac{Li-1}{3}$$

$$\text{TMI after component score} = 100 - \left[\frac{100}{2}\right] \sum_{i=1}^{2} \frac{Li-1}{3}$$

3. Calculation of the joint utility scores

Computation of the joint utility score of breast MRI after diagnostic mammography followed Thompson et al [5] (refer to Appendix 1 of that paper), and utilized the three most common methods of estimating joint utility, namely additive, multiplicative and minimum models. For breast MRI and mammography TMI summated scale scores, the general equation for the additive model is:

$$U_{Additive} = 100 - [(100 - TMI_{MRI}) + (100 - TMI_{Mammo})]$$

The general equation for the multiplicative model is:

$$U_{Multiplicative} = 100 \times \frac{TMI_{MRI}}{100} \times \frac{TMI_{Mammo}}{100}$$

The general equation for the minimum model is:

$$U_{Minimum} = \text{minimum of } (TMI_{MRI}, TMI_{Mammo})$$

4. Sensitivity analysis incorporating multiple imputation for missing covariate data

Using complete case analysis with listwise deletion resulted in 16% of records being dropped from the multivariable regression models (Table 3 of the main paper). To assess the impact of missing data on the reported multivariable models, multiple imputation by chained equations [6,7], otherwise known as fully conditional specification, was used to impute missing covariate data for the N=244 subjects with TMI summated scale scores available for both modalities. A key assumption of multiple imputation is that the data are missing at random (MAR); or, that the probability of data being missing does not depend on the unobserved data, conditional on the observed data. Separate imputations were performed for the multivariable breast MRI TMI summated scale score regression model, and the multivariable joint utility score regression models.

For the breast MRI TMI summated scale score, the imputation model included the response variable (TMI summated scale score), as well as all covariates from the analysis model (age, race, ethnicity, insurance status, PROMIS-10 physical and mental T scores [T0], the revised 5-item ASC cancer worry subscale score [T0], and decision autonomy preference [T0]). In addition, given that the PROMIS-10 physical and mental T scores at T0 had the most missing data, the PROMIS-10 physical and mental T score collected at the first post-op visit following breast surgery (time point T2) were added to the imputation model as auxiliary variables (correlation=0.61 for physical T score between T0 and T2, and 0.67 for mental T score between T0 and T2); the mammography TMI summated scale score was also added as an auxiliary variable. In the imputations, a regression model was used for continuous variables, and a discriminant function model was used for categorical variables. Imputations were performed using SAS 9.4 PROC MI, with a total of 50 imputation samples. The same multivariable model was then fit to each imputation sample, with parameter estimates combined across samples using Rubin's rules [8] via SAS 9.4 PROC MIANALYZE.

For the joint utility scores, the imputation model included the response variables for the three models (joint (additive) utility score, joint (multiplicative) utility score, and joint (minimum) utility score), as well all covariates from the analysis models (age, race, ethnicity, insurance status, PROMIS-10 physical and mental T scores [T0], the revised 5-item ASC cancer worry

© 2021 Fazeli S et al. *JAMA Network Open.*

subscale score [T0], and decision autonomy preference [T0]). In addition, given that the PROMIS-10 physical and mental T scores at T0 had the most missing data, the PROMIS-10 physical and mental T score collected at T2 were again added to the imputation model as auxiliary variables; the breast MRI TMI summated scale score was also added as an auxiliary variable. In the imputations, a regression model was used for continuous variables, and a discriminant function model was used for categorical variables. Imputations were performed using SAS 9.4 PROC MI, with a total of 50 imputation samples. The same multivariable models were then fit to each imputation sample, with parameter estimates combined across samples using Rubin's rules [8] via SAS 9.4 PROC MIANALYZE.

The amount of missing data by covariate is as follows:

**Variable**	**Amount missing**	**Percent missing**
Age	0/244	0%
Race	0/244	0%
Ethnicity	0/244	0%
Insurance Status	0/244	0%
Decision Autonomy Preference	0/244	0%
PROMIS-10 mental T Score	32/244	13%
PROMIS-10 physical T Score	31/244	13%
Revised 5-item ASC: cancer subscale	3/244	1%
Mammography TMI summated scale score	0/244	0%
Breast MRI TMI summated scale score	0/244	0%
Joint utility (additive) score	0/244	0%
Joint utility (multiplicative) score	0/244	0%
Joint utility (minimum) score	0/244	0%

© 2021 Fazeli S et al. *JAMA Network Open.*

eTable 1 – Summary of univariable associations between the breast MRI TMI summated scale score and prespecified covariates.

Variable	Level	Breast MRI TMI summated scale score (N=244)		
		**N**	**Estimate [1]**	**p-value [2]**
**Age**		244	0.13 (0.07) (R^2=0.02)	**0.049 ***
**Race**	Black/African American	30	83.5 (12.0)	0.32
	Other	17	84.6 (11.4)	
	White	197	86.4 (10.3)	
**Ethnicity**	Hispanic	9	86.8 (10.3)	0.81
	Non-Hispanic/Unknown	235	85.9 (10.6)	
**Insurance status [3]**	Private insurance	188	85.4 (10.7)	0.37
	Medicare/Other government insurance	48	87.6 (9.5)	
	Medicaid/Uninsured	8	88.1 (14.4)	
**Decision autonomy preference**	My surgeon should make the decision with little input from me/My surgeon should make the decision but seriously consider my opinion	21	85.7 (10.5)	0.16
	My surgeon and I should make the decision together	174	86.7 (10.0)	
	I should make the decision after seriously considering my surgeon's opinion	49	83.4 (12.5)	
**Revised 5-item ASC: cancer subscale [4]**		241	-2.79 (0.82) (R^2=0.05)	**<0.001 *****
**PROMIS-10 Physical T score [4]**		213	0.20 (0.09) (R^2=0.02)	**0.03 ***
**PROMIS-10 Mental T score [4]**		212	0.32 (0.10) (R^2=0.04)	**0.002 ****

[1] For continuous variables, the estimate and standard error are the slope and standard error from a univariable linear regression model. For categorical variables, group means and standard deviations are provided.

[2] For continuous variables, the p-value is from a univariable linear regression model. For categorical variables, the p-value is from a one-way ANOVA model.

[3] Used as a proxy for socioeconomic status. No other variables of a similar nature were collected.

[4] The amount of missing covariate data is as follows:

Revised 5-item ASC cancer subscale: N=3 (1%)
PROMIS-10 Physical T score: N=31 (13%)
PROMIS-10 Mental T score: N=32 (13%)

*** <0.05 ** <0.01 *** <0.001**

© 2021 Fazeli S et al. *JAMA Network Open.*

eTable 2 – Summary of univariable associations between the joint utility scores and prespecified covariates.

Variable	Level	Joint utility score (Additive model)			Joint utility score (Multiplicative model)			Joint utility score (Minimum model)		
		N	Estimate [1]	p-value [2]	N	Estimate [1]	p-value [2]	N	Estimate [1]	p-value [2]
**Age**		244	0.22 (0.10) (R^2=0.02)	**0.03 ***	244	0.17 (0.09) (R^2=0.02)	**0.047 ***	244	0.14 (0.07) (R^2=0.02)	**0.04 ***
**Race**	Black/African American	30	75.1 (17.0)	0.70	30	76.8 (15.0)	0.65	30	81.9 (11.8)	0.38
	Other	17	73.1 (14.5)		17	75.0 (12.8)		17	81.8 (9.3)	
	White	197	76.3 (15.7)		197	78.0 (13.5)		197	84.2 (10.1)	
**Ethnicity**	Hispanic	9	76.2 (15.1)	0.95	9	77.9 (13.2)	0.96	9	84.7 (8.5)	0.79
	Non-Hispanic/Unknown	235	75.9 (15.8)		235	77.6 (13.6)		235	83.7 (10.3)	
**Insurance status [3]**	Private insurance	188	75.0 (16.3)	0.27	188	76.9 (13.9)	0.30	188	83.4 (10.5)	0.47
	Medicare/Other government insurance	48	79.0 (13.3)		48	80.2 (12.0)		48	85.3 (9.2)	
	Medicaid/Uninsured	8	78.6 (15.3)		8	79.5 (13.9)		8	82.1 (11.3)	
**Decision autonomy preference**	My surgeon should make the decision with little input from me/My surgeon should make the decision but seriously consider my opinion	21	74.6 (14.9)	0.41	21	76.4 (13.1)	0.42	21	82.8 (9.9)	0.49
	My surgeon and I should make the decision together	174	76.7 (14.9)		174	78.3 (13.1)		174	84.2 (9.5)	
	I should make the decision after seriously considering my surgeon's opinion	49	73.5 (18.8)		49	75.6 (15.4)		49	82.4 (12.6)	
**Revised 5-item ASC: cancer subscale [4]**		241	-6.6 (1.2) (R^2=0.12)	**<0.001 *****	241	-5.7 (1.0) (R^2=0.12)	**<0.001 *****	241	-3.5 (0.8) (R^2=0.08)	**<0.001 *****
**PROMIS-10 Physical T score [4]**		213	0.37 (0.13) (R^2=0.04)	**0.006 ****	213	0.33 (0.12) (R^2=0.04)	**0.005 ****	213	0.23 (0.09) (R^2=0.03)	**0.009 ****
**PROMIS-10 Mental T score [4]**		212	0.52 (0.15) (R^2=0.06)	**<0.001 *****	212	0.47 (0.13) (R^2=0.06)	**<0.001 *****	212	0.32 (0.10) (R^2=0.05)	**0.001 ****

[1] For continuous variables, the estimate and standard error are the slope and standard error from a univariable linear regression model. For categorical variables, group means and standard deviations are provided.

[2] For continuous variables, the p-value is from a univariable linear regression model. For categorical variables, the p-value is from a one-way ANOVA model.

[3] Used as a proxy for socioeconomic status. No other variables of a similar nature were collected.

[4] The amount of missing covariate data is as follows:

Revised 5-item ASC cancer subscale: N=3 (1%)
PROMIS-10 Physical T score: N=31 (13%)
PROMIS-10 Mental T score: N=32 (13%)

*** <0.05 ** <0.01 *** <0.001**

© 2021 Fazeli S et al. *JAMA Network Open.*

**eTable 3**: Multivariable regression model for the joint utility score (multiplicative model) of a testing sequence of diagnostic mammography followed by breast MRI in DCIS.				
**Joint utility (multiplicative) score model** [1]	**Complete case estimates (N=206)** **R^2=0.20**		**Multiple imputation estimates (N=244)** [2] **R^2=0.17**	
	**Parameter Estimate (SE)**	**P-value**	**Parameter Estimate (SE)**	**P-value**
**Intercept** [3]	75.90 (2.06)	<0.001	76.22 (1.97)	<0.001
**Age**	0.03 (0.10)	0.77	0.03 (0.10)	0.77
**Revised 5-item ASC: cancer subscale**	-5.89 (1.14)	**<0.001 *** **	-5.44 (1.12)	**<0.001 *** **
**PROMIS-10 Physical T score**	0.23 (0.13)	0.08	0.27 (0.15)	0.08
**PROMIS-10 Mental T score**	0.17 (0.16)	0.29	0.13 (0.18)	0.48
**Race:** Black/African American (vs. White)	-3.21 (2.57)	0.21	-1.73 (2.54)	0.50
**Race:** Other (vs. White)	-0.46 (3.51)	0.90	-0.77 (3.51)	0.83
**Ethnicity:** Hispanic (vs. Non-Hispanic/Unknown)	-0.10 (4.80)	0.98	-0.45 (4.65)	0.92
**Insurance status**: Medicare/Other government insurance vs. private insurance	1.04 (2.47)	0.68	0.64 (2.37)	0.79
**Insurance status:** Medicaid/Uninsured vs. private insurance	0.53 (4.60)	0.91	0.42 (4.75)	0.93
**Decision autonomy preference:** Surgeon-driven (vs. Patient-driven)	-0.91 (3.49)	0.79	-0.96 (3.43)	0.78
**Decision autonomy preference:** Shared (vs. Patient-driven)	2.32 (2.23)	0.30	2.43 (2.11)	0.25

[1] Parameter estimates for continuous covariates are interpreted as the change in mean response per 1-unit increase. Parameter estimates for categorical covariates are interpreted as the difference in mean response in comparison to the reference level.

[2] Multiple imputation for missing covariate data was performed for the subset of patients with available TMI summated scale scores for both modalities (N=244, refer to Figure 1 of the main paper).

[3] Continuous covariates were centered. Thus, the intercept can be interpreted as the mean joint utility score for patients who are at the mean age (59.1 years), mean ASC cancer worry level (2.41), mean physical and mental T scores (52.40 and 51.76, respectively), and who are at the reference level of the categorical covariates (White, non-Hispanic, private insurance, patient-driven decision preference).

SE=Standard error
R^2=Coefficient of determination from the multivariable linear regression model

*** <0.05 ** <0.01 *** <0.001**

© 2021 Fazeli S et al. *JAMA Network Open.*

**eTable 4**: Multivariable regression model for the joint utility score (minimum model) of a testing sequence of diagnostic mammography followed by breast MRI in DCIS.				
**Joint utility (minimum) score model [1]**	**Complete case estimates (N=206)**<br><br>**R^2=0.17**		**Multiple imputation estimates (N=244) [2]**<br><br>**R^2=0.13**	
	**Parameter Estimate (SE)**	**P-value**	**Parameter Estimate (SE)**	**P-value**
**Intercept [3]**	83.25 (1.60)	<0.001	83.30 (1.52)	<0.001
**Age**	0.07 (0.08)	0.35	0.06 (0.08)	0.41
**Revised 5-item ASC: cancer subscale**	-3.48 (0.89)	**<0.001 *** **	-3.21 (0.86)	**<0.001 *** **
**PROMIS-10 Physical T score**	0.16 (0.10)	0.12	0.17 (0.11)	0.13
**PROMIS-10 Mental T score**	0.15 (0.12)	0.23	0.11 (0.14)	0.42
**Race:** Black/African American (vs. White)	-3.96 (2.00)	**0.049 ***	-2.66 (1.97)	0.18
**Race:** Other (vs. White)	-0.87 (2.73)	0.75	-0.83 (2.71)	0.76
**Ethnicity:** Hispanic (vs. Non-Hispanic/Unknown)	2.87 (3.73)	0.44	1.40 (3.59)	0.70
**Insurance status**: Medicare/Other government insurance vs. private insurance	0.05 (1.92)	0.98	-0.27 (1.83)	0.88
**Insurance status:** Medicaid/Uninsured vs. private insurance	-3.54 (3.57)	0.32	-3.16 (3.68)	0.39
**Decision autonomy preference:** Surgeon-driven (vs. Patient-driven)	-1.01 (2.71)	0.71	-0.60 (2.65)	0.82
**Decision autonomy preference:** Shared (vs. Patient-driven)	1.53 (1.73)	0.38	1.46 (1.63)	0.37

[1] Parameter estimates for continuous covariates are interpreted as the change in mean response per 1-unit increase. Parameter estimates for categorical covariates are interpreted as the difference in mean response in comparison to the reference level.
[2] Multiple imputation for missing covariate data was performed for the subset of patients with available TMI summated scale scores for both modalities (N=244, refer to Figure 1 of the main paper).
[3] Continuous covariates were centered. Thus, the intercept can be interpreted as the mean joint utility score for patients who are at the mean age (59.1 years), mean ASC cancer worry level (2.41), mean physical and mental T scores (52.40 and 51.76, respectively), and who are at the reference level of the categorical covariates (White, non-Hispanic, private insurance, patient-driven decision preference).

SE=Standard error
R^2=Coefficient of determination from the multivariable linear regression model

*** <0.05 ** <0.01 *** <0.001**

© 2021 Fazeli S et al. *JAMA Network Open.*

eReferences

1. Swan JS, Kong CY, Lee JM, et al. Patient and Societal Value Functions for the Testing Morbidities Index. *Med Decis Mak*. 2013;33(6):819-838. doi:10.1177/0272989X13487605
2. Shannon Swan J, Yin Kong C, Hur C, et al. Comparing Morbidities of Testing With a New Index: Screening Colonoscopy Versus Core-Needle Breast Biopsy. *J Am Coll Radiol*. 2015;12:295-301. doi:10.1016/j.jacr.2014.08.014
3. Sakala MD, Carlos RC, Mendiratta-Lala M, Quint EH, Maturen KE. Understanding Patient Preference in Female Pelvic Imaging. *Acad Radiol*. 2018;25(4):439-444. doi:10.1016/j.acra.2017.10.011
4. Shannon Swan J, Hur C, Lee P, Motazedi T, Donelan K. Responsiveness of the Testing Morbidities Index in Colonoscopy. *Value Heal*. 2013;16:1046-1053. doi:10.1016/j.jval.2013.07.008
5. Thompson AJ, Sutton M, Payne K. Estimating Joint Health Condition Utility Values. *Value Heal*. 2019;22(4):482-490. doi:10.1016/j.jval.2018.09.2843
6. van Buuren S. Multiple imputation of discrete and continuous data by fully conditional specification. *Stat Methods Med Res*. 2007;16(3):219-242. doi:10.1177/0962280206074463
7. Raghunathan T, Lepkowski J, Hoewyk J Van, Solenberger P. A multivariate technique for multiply imputing missing values using a sequence of regression models. *Surv Methodol*. 2001;27:85-95.
8. Little RJA, Rubin DB. *Statistical Analysis with Missing Data*. 2nd ed. Hoboken, NJ: Wiley; 2002.

© 2021 Fazeli S et al. *JAMA Network Open.*
